# Rationale, design and conduct of a comprehensive evaluation of a primary care based intervention to improve the quality of life of osteoarthritis patients. The PraxArt-project: a cluster randomized controlled trial [ISRCTN87252339]

**DOI:** 10.1186/1471-2458-5-77

**Published:** 2005-07-19

**Authors:** Thomas Rosemann, Thorsten Körner, Michel Wensing, Jochen Gensichen, Christiane Muth, Stefanie Joos, Joachim Szecsenyi

**Affiliations:** 1 Department. of General Practice and Health Services Research, University of Heidelberg, Voßstr. 2, 69115 Heidelberg, Germany; 2 Centre for Quality of Care Research, Radboud University Medical Centre Nijmegen, P.O. Box 9101, 6500 HB Nijmegen, The Netherlands; 3 Institute for General Practice, Chronic Care and Health Services Research Unit, University of Frankfurt, Theodor-Stern-Kai 7, 60590 Frankfurt a.M. Germany

## Abstract

**Background:**

Osteoarthritis (OA) has a high prevalence in primary care. Conservative, guideline orientated approaches aiming at improving pain treatment and increasing physical activity, have been proven to be effective in several contexts outside the primary care setting, as for instance the Arthritis Self management Programs (ASMPs). But it remains unclear if these comprehensive evidence based approaches can improve patients' quality of life if they are provided in a primary care setting.

**Methods/Design:**

PraxArt is a cluster randomised controlled trial with GPs as the unit of randomisation. The aim of the study is to evaluate the impact of a comprehensive evidence based medical education of GPs on individual care and patients' quality of life. 75 GPs were randomised either to intervention group I or II or to a control group. Each GP will include 15 patients suffering from osteoarthritis according to the criteria of ACR.

In intervention group I GPs will receive medical education and patient education leaflets including a physical exercise program. In intervention group II the same is provided, but in addition a practice nurse will be trained to monitor via monthly telephone calls adherence to GPs prescriptions and advices and ask about increasing pain and possible side effects of medication.

In the control group no intervention will be applied at all. Main outcome measurement for patients' QoL is the GERMAN-AIMS2-SF questionnaire. In addition data about patients' satisfaction (using a modified EUROPEP-tool), medication, health care utilization, comorbidity, physical activity and depression (using PHQ-9) will be retrieved.

Measurements (pre data collection) will take place in months I-III, starting in June 2005. Post data collection will be performed after 6 months.

**Discussion:**

Despite the high prevalence and increasing incidence, comprehensive and evidence based treatment approaches for OA in a primary care setting are neither established nor evaluated in Germany. If the evaluation of the presented approach reveals a clear benefit it is planned to provide this GP-centred interventions on a much larger scale.

## Background

Arthritis is a most frequent affection of joints and a common condition in general practice (roughly 60–80 patients per thousand cases with an average of 5.7 GP contacts per quarter) [1,2]. The GP is the primary contact for arthritis patients and the main care provider for most patients. Previous studies, including our own qualitative pilot study, have shown that arthritis related pain and fear of increasingly reduced mobility represent the most important burden for arthritis patients [3]. There was a large general need for information among patients concerning as for instance individual options to influence the course of the disease [3,4]. GPs approaches to OA varied widely and patient education concerning life style and motivation for physical activity was mostly vague and unsighted [5]. An important need for information about evidence based pain management according to WHO-recommendations was detected among GPs. Several studies underlined the effectiveness of complex interventions with active patient involvement such as the "Arthritis-self-management-programs" (ASMPs) in the US and Canada. However, these programs generally take place outside of medical care settings [6-9]. But even there is quite good evidence for these interventions, the implementation of these approaches in a primary care setting seems to be accompanied with additional problems, while in these setting less positive results were revealed in former studies [10-12].

However, it still remains unclear what approach is the best to implement evidence based treatment approaches into daily practice [13]. Without a doubt different settings and cultures of implementing knowledge have to be considered. In Germany quality circles are a well established concept and several studies have proven their impact on different outcome parameters as for instance on prescriptions [14]. But previous studies revealed that the improvement in care is mostly moderate if no additional strategies are provided to improve the impact of the meetings for GPs [13]. Regarding the field of chronic care and especially degenerative joint diseases, the involvement of practice nurses, as for instance to perform frequent telephone calls, has shown to increase the patients' quality of life [15]. It could be assumed that the impact on patients may increase if the induced implementations on the patients' level are frequently monitored by practice nurses.

## Methods

### Aim of the study

The study examines whether a multifaceted intervention with evidence based medical education for GPs can improve the quality of life of arthritis patients.

#### Scientific hypothesis

A targeted evidence based medical education for GPs on osteoarthritis has no effect on the quality of life of patients with degenerative joint diseases and their prescribed medication. Monitoring GPs' prescriptions and advices for lifestyle changes by monthly telephone calls of practice assistants with a structured form is not superior.

The study content is guided by internationally available evidence for arthritis therapy in General Practice. Due to the lack of a German arthritis guideline an evidence based review for arthritis care in General Practice will be compiled from European guidelines [7,9,16]. Subsequently a preliminary guideline will be elaborated based on this material. Additionally motivational strategies and communication skills will be taught to GPs in order to improve the implementation of life style changes.

### Study design

The study is a (prospective) cluster-randomized, open, three-armed intervention study. The design of a cluster randomized study was chosen because this has optimal internal validity (absence of confounders) while avoiding contamination of interventions associated with patient randomization.

### Sample size

Sample size calculations for cluster randomized trials differ completely from sample size calculations for common RCTs [17-19]. Based on the main outcome parameter (QoL) and the main outcome-assessment instrument (GERMAN-AIMS2-SF) [20], we performed a power calculation with the Cluster Randomization Sample Size Calculator ver.1.02 of the University of Aberdeen. Assuming an effect size of 30 % (according to recommendations of Guillemin et al. [21]), an ICC of 0.03 (based on previous studies and on data available at the website of the University of Aberdeen [22]) a power of 90 % a mean of 2.7 and a minimal difference to detect of 0.9 and a significance level of 0.05, we have to include 14 patients in each of the 25 practices.

### Recruitment of GPs and randomization

The GPs are the unit of randomization. They were eligible for randomization if their practice had a contract with all German insurances, so it is assured that patients of all social levels have unlimited admission to the practice. If they were working in a non-single-handed practice, it was important that they had their own patients which could clearly be allocated to them. In Germany most of GPs work in single handed practices, but even if not, patients frequently are treated by one specific GP in a practice, so that these inclusion criteria will not represent a source of bias, because only an absolute minority of practices will not be eligible for inclusion due to these criteria. About 500 GPs in the area of Baden-Wuerttemberg and Bavaria, fulfilling inclusion criteria, were invited by a formal letter of the Department of General Practice and Health services Research of the University of Heidelberg, to participate in the study. 120 GPs gave their written consent to participate in the study. Based on detailed information about the practice and the GP, the inclusion criteria were checked. No GP or no practice had to be excluded due to the inclusion criteria. The 120 GPs were invited to information meetings were the aim and the procedure of the study were explained in detail. After the meeting, the 120 eligible GPs were put on a list with numbers from 1 to 120. Out of this list, 75 GPs were randomized with SPSS version 11.0 to one of the intervention groups or the control group by an independent assistant who is not familiar to one of the participating doctors.

### Patient inclusion criteria

To be eligible for inclusion patients have to be adult and diagnosed with gonarthritis or coxarthritis according to the ACR criteria [23]. They will be identified by the following ICD-10 code in patients file: M 16.0–16.9 and M 17.0–17.5. Based on this process, participating practices keep an alphabetic record of their patients. Patients from this list are contacted in consecutive order of appearance in the practice and informed about the option to participate in the study. After giving their written informed consent they receive the questionnaire and a stamped envelope with the postal address of the university. The patients are asked to return this questionnaire in the envelope to the university. Neither the GP nor the practice team has any possibility to get knowledge of the patients' answers.

### Patient exclusion criteria

1. Insufficient German language skills.

2. Patients, who contacted the practice for emergencies only or as a substitute practice.

### Data collection

After giving their informed and written consent to participate in the study patients will receive a questionnaire which is based on arthritis-related indicators and include the GERMAN-AIMS2-SF [20], a revised version of the EUROPEP-questionnaire[24], as well as items that assess secondary outcome parameters as shown in table 1. The envelopes are opened at the university by an independent research assistant and immediately scanned with the "eyes and hands ™ FORMS"-Software (Version 5) of Read Soft. A TIF-file is generated out of each questionnaire to avoid any data-manipulation and to ease data storage. The data are transferred into the SPSS program (version 12.0). Patients' information on medication and health care utilization will be checked by three research assistants, visiting each practice.

**Table 1:** Outcome-parameters and instruments of the study

**Outcome-Parameter (Patient)**	**Instrument**
Primary Outcome	
Quality of life	GERMAN-AIMS2-SF
**Secondary outcome**	
Health care utilization	questonnaire, retrospective chart review
Patient satisfaction	modified EUROPEP
Physical activity	6 minutes walking, CDC-criteria, specific questions
Medication	questionnaire, retrospective chart review
**Confounder control**	
Mental comorbidity	PHQ-9

### Outcome-Parameter

Table 1 displays the outcome parameters and associated used instruments. The primary outcome is quality of life assessed by the AIMS2-SF questionnaire, an internationally validated instrument for the assessment of quality of life among arthritis patients [21]. We have validated this instrument for German general practice in a previous study [20].

#### Secondary outcomes include

• Medication (evidence based use of NSAR, application of WHO-recommendations); data retrieved from patients chart

• Health Care utilization (referrals to orthopedists, imaging, inpatient care, physiotherapy); data retrieved from patients chart

• Physical activity (percentage of patients meeting CDC criteria)

• Patient satisfaction (modified EUROPEP-questionnaire) [25]

• Potential confounders are being detected (concurrent depression may influence the potential motivational change for more physical activity) by means of PHQ-9 [26].

These data will be compiled from patient questionnaires and patients chart review. All instruments represent well established and validated instruments. Measurements and analysis will take place before intervention (pre-data-collection) and 6 months later (post-data collection).

### Intervention

#### 1. Implementation strategy – aiming at the GP

The implementation strategy consists of two interactive quality circle meetings of 3 hours including 12–13 participating GPs (Intervention group I). These meetings have three main contents: evidenced based treatment of osteoarthritis in a primary care setting, optimizing pain treatment according to the WHO recommendations, providing advanced motivation skills. Intervention group II represents an "add-on" approach. GPs will participate in meetings with the same content as GPs of intervention group I. In addition to these meetings, practice assistants will also participate in a course. During this course the assistance is trained to call patients and provide an OA specific telephone questionnaire which addresses to three main topics: Side effects of the prescribed drugs, adherence to recommended physical activity and changes in pain.

In both intervention groups, GPs will receive a summary of evidence based treatments of OA in a primary care setting. These information contains the recommendations of the EULAR group for the treatment of OA and the information provided by the German Medical Association [7,9,16]. GPs will also receive two written patient leaflets. Leaflet one provides information about the cause and the treatment possibilities as well as coping strategies and contact addresses of self help groups for the patients. Leaflet two provides a detailed exercise program, developed by a German self help group, the "Deutsche Rheumaliga". This leaflet contains pictures and a step by step exercise program, even patients with severe OA can perform.

#### 2. Clinical intervention

In intervention group II frequent telephone calls will be provided by each practice. For this purpose an osteoarthritis specific telephone questionnaire – the "ArthMol" tool – has been developed in cooperation with the Department of General Practice at Johann-Wolfgang Goethe-University Clinic, Frankfurt am Main. GPs' assistants will contact OA patients via telephone every four weeks and complete a structured telephone form during the conversation. The form contains items referring to pain, adherence to prescriptions, the exercise program and possible side effects of the medication. According to the urgency of the information it is either directly reported to the GP or transmitted during the following day.

There is no implementation strategy in the control group (group III).

### Timeframe of the study

The study team has already randomized the 75 out of the initial recruited 120 GPs who have declared their willingness to participate in the study and to accepted random assignment to the different groups. The patient inclusion and pre data collection will take place in months I-III, the intervention (quality circles and telephone calls in group II) will take place in months IV-X. Post data collection will be performed in months X-XII.

### Description of risks

Serious risks or undesired effects of questionnaires have not been described in the literature. There are no specific risks related to the study.

### Ethical and legal aspects

#### Ethical principles

The study is being conducted in accordance with medical professional codex and the Helsinki Declaration as of 1996 as well as the German Federal Data Security Law (BDSG).

Study participation of patients is voluntary and can be cancelled at any time without provision of reasons and without negative consequences for their future medical care.

#### Patient informed consent

Previous to study participation patients receive written and spoken information about the content and extent of the planned study; for instance about potential benefits for their health and potential risks. In case of acceptance they sign the informed consent form.

In case of study discontinuation all material will be destroyed or the patient will be asked if he/she accepts that existing material can be analyzed in the study.

### Legal principles

#### Vote of the ethics committee

The study protocol was approved by the ethics committee of the University of Heidelberg previous to the start of the study in January 2005. Inclusion of patients/ participants did not start unless there was a written and unrestricted positive vote of the ethics committee. This vote was received in March 2005 (approval number 021/2005).

#### Data security/ disclosure of original documents

The patient names and all other confidential information fall under medical confidentiality rules and are treated according to German Federal Data Security Law (BDSG). The results of the patient questionnaires are not accessible to the GPs. Questionnaires are directly mailed to the study center by the patient.

All study related data and documents are stored on a protected central server of the Heidelberg University Clinic. Only direct members of the internal study team can access the respective files.

Intermediate and final reports are stored in the office of the Department of General Practice and Health Services Research at the Heidelberg University Clinic.

## Competing interests

The author(s) declare that they have no competing interests.

## Authors' contributions

TR, TK and MW conceived and performed the study and draft the manuscript. JG and CM developed the "ArthMoL"-tool. SJ and JS participated in the study design. All authors read and approved the final manuscript.

**Figure 1:**
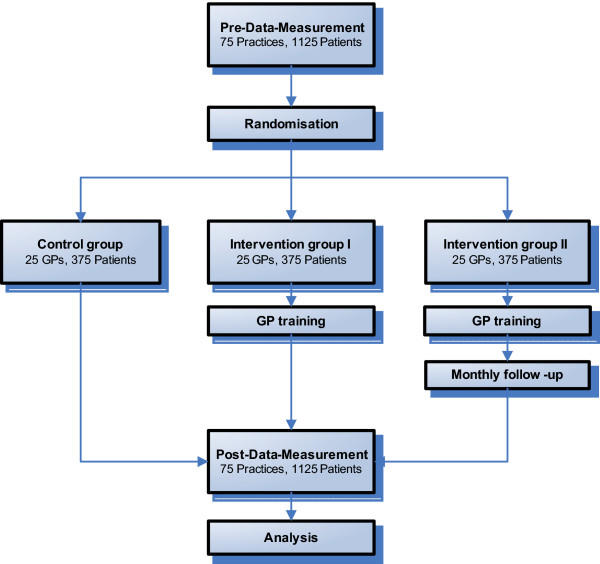
Flow diagram of the progress through the phases of the study.

## Pre-publication history

The pre-publication history for this paper can be accessed here:


